# Effect of cryopreservation on DNA damage and DNA repair activity in human blood samples in the comet assay

**DOI:** 10.1007/s00204-021-03012-4

**Published:** 2021-03-05

**Authors:** Ezgi Eyluel Bankoglu, Franzisca Stipp, Johanna Gerber, Florian Seyfried, August Heidland, Udo Bahner, Helga Stopper

**Affiliations:** 1 Institute of Pharmacology and Toxicology, University of Wuerzburg, Versbacher Straße 9, 97078 Wuerzburg, Germany; 2 Department of General and Visceral, Vascular and Pediatric Surgery, University Hospital Wuerzburg, Wuerzburg, Germany; 3 Department of Internal Medicine and KfH Kidney Center, University of Wuerzburg, KfH Kidney Center Wuerzburg, Wuerzburg, Germany

**Keywords:** DNA damage, DNA repair, Comet assay, Blood samples, Human biomonitoring

## Abstract

The comet assay is a commonly used method to determine DNA damage and repair activity in many types of samples. In recent years, the use of the comet assay in human biomonitoring became highly attractive due to its various modified versions, which may be useful to determine individual susceptibility in blood samples. However, in human biomonitoring studies, working with large sample numbers that are acquired over an extended time period requires some additional considerations. One of the most important issues is the storage of samples and its effect on the outcome of the comet assay. Another important question is the suitability of different blood preparations. In this study, we analysed the effect of cryopreservation on DNA damage and repair activity in human blood samples. In addition, we investigated the suitability of different blood preparations. The alkaline and FPG as well as two different types of repair comet assay and an in vitro hydrogen peroxide challenge were applied. Our results confirmed that cryopreserved blood preparations are suitable for investigating DNA damage in the alkaline and FPG comet assay in whole blood, buffy coat and PBMCs. Ex vivo hydrogen peroxide challenge yielded its optimal effect in isolated PBMCs. The utilised repair comet assay with either UVC or hydrogen peroxide-induced lesions and an aphidicolin block worked well in fresh PBMCs. Cryopreserved PBMCs could not be used immediately after thawing. However, a 16-h recovery with or without mitotic stimulation enabled the application of the repair comet assay, albeit only in a surviving cell fraction.

## Introduction

Human biomonitoring is increasingly appreciated due to its relevance to public health. To study human exposure and effects of occupational or environmental factors on human health, mostly blood preparations are used because of relatively easy availability (Azqueta et al. 2020). Increased DNA damage in blood derived cells has been found to correlate with increased risk of cancer and degenerative diseases (Bonassi et al. 2007; Møller et al. 2020). DNA repair can reduce genomic damage, or in the case or erroneous repair or overwhelmed repair enzymes lead to its manifestation (Langie et al. 2015). Therefore, DNA damage and DNA repair activity are both important endpoints to be assessed. In addition, these parameters can be used to study individual DNA damage or repair levels, which can be influenced by life style factors. While the latter can more easily be done with freshly obtained samples, studies regarding occupational or environmental influence on DNA integrity require large sample numbers, which have to be collected over an extended time period. Only assays, which allow the use of stored samples are suitable for such studies.

The comet assay is a simple technique, which offers with its several modified versions a useful tool for human biomonitoring (Collins and Azqueta 2012a). It allows to determine and characterise DNA damage and DNA repair activity. One of the main challenges is working with large sample numbers. To overcome this issue, an enhanced throughput version can be used (Azqueta et al. 2013; Gutzkow et al. 2013). Another problem in large studies is that samples are collected on different days at different locations and must be stored. For the comet assay, they must be processed simultaneously. The storage of human blood cells is commonly done as frozen whole blood or buffy coat preparations. It has been shown already that frozen whole blood preparations can be used in the comet assay if they were frozen in a suitable manner, which is not yet the case for many long-existing biobanks and sample collections (Al-Salmani et al. 2011; Koppen et al. 2017; Milić et al. 2019). Some recent studies investigated the effect of cryopreservation on DNA damage in different blood preparations (Ladeira et al. 2019). However, these studies are mostly limited to DNA damage and do not assess DNA repair activity. Furthermore, sample numbers are small. There is still a need for investigation of the suitability of frozen buffy coat or peripheral blood mononuclear cells (PBMC) with a bigger sample size. Furthermore, the applicability of protocol variations for characterisation of the DNA damage and DNA repair activity still needs to be established. DNA repair is an important parameter for susceptibility to accumulations of mutations and cancer risk. Individual differences in DNA repair are sometimes analysed by measuring expression of repair genes, but the activity of repair proteins, which can only be assessed in a functional assay, is not usually reported in biomonitoring type studies (Azqueta et al. 2019; Collins and Azqueta 2012b). This is partly due to the lack of well-established methods. The comet assay offers a good alternative with its various modified forms for measuring DNA repair activity, although further understanding is necessary. In this study, we aimed to fill this gap by investigating the use of cryopreserved PBMCs in the aphidicolin block repair comet assay. For this, we studied the effect of cryopreservation, using different blood preparations and various modified versions of the comet assay to assess DNA damage and DNA repair activity.

## Materials and methods

### Materials

GelRed was from Biotrend (Köln, Germany). Normal melting point agarose, dimethyl sulfoxide (DMSO) and sodium hydroxide were from Carl Roth (Karlsruhe, Germany). RPMI 1640 medium, l-glutamine, Triton X-100, pyhtohaemaglutinine, histopaque, methyl methanesulfonate, potassium bromate and low melting point agarose were from Sigma-Aldrich (Steinheim, Germany). Formamidopyrimidine-DNA glycolase of Escherischia coli (FPG protein) was isolated from *E. coli* strain JM105, carrying the plasmid pFPG230 (kindly supplied by Prof. Dr. Bernd Epe, University of Mainz, Germany).

### Methods

#### Sample preparation

The study was intended to investigate the effect of cryopreservation on DNA damage and DNA repair in different blood preparations. For this aim, we used 20 anonymized blood samples. These were remaining blood volumes from studies with University Wuerzburg ethical approval numbers 186/14 and 202/19, partially published in (Bankoglu et al. 2020, 2018), which were used fresh and also cryopreserved as whole blood or isolated peripheral blood mononuclear cells. Whole blood samples were aliquoted as 250 µl portions and frozen without cryoprotectant. For buffy coat, 1 ml of blood was centrifuged at 200 *g* for 10 min at room temperature. The buffy coat layer between erythrocytes and plasma was collected with a Pasteur pipette. The isolated buffy coat was cryopreserved as 250-µl aliquots without cryoprotectant. Isolation of peripheral blood mononuclear cells (PBMCs) was done using 3-ml blood diluted with PBS. This solution was layered on an equal amount of histopaque and then centrifuged at room temperature at 535 *g* for 30 min. After centrifugation, the PBMC layer was collected with the help of a Pasteur pipette and washed twice with medium (RPMI 1640 containing 10% fetal calf serum and 1% l-glutamin) at room temperature at 300 *g* for 10 min. Finally, PBMCs were resuspended in 3 ml medium and the cell number was determined using a cell counting chamber. Samples were cryopreserved by diluting 2 × 10^6^ cells/ml freezing medium (RPMI 1640 medium containing 10% fetal calf serum, 1% _L_-glutamin and 10% DMSO). Aliquots of whole blood, buffy coat and isolated PBMCs were frozen overnight in a freezing container with isopropanol at − 80 °C and stored at − 80 °C.

For thawing samples, we used a 37 °C water bath. Whole blood and buffy coat samples were then placed on ice until use. PBMCs were centrifuged at 4 °C, 750 *g* for 5 min and then the cell pellet was diluted in PBS to reach the desired cell density and kept on ice.

#### Exogenous DNA damage induction

An ex vivo hydrogen peroxide treatment (40 µM at 4 °C for 5 min) was performed for all blood preparations to investigate the effect of cryopreservation as well as to compare the sensitivity differences between the blood preparations.

In addition, 40 µM hydrogen peroxide (H_2_O_2_) was used to induce oxidative DNA lesions to determine the base excision repair (BER) activity. An UVC treatment (5 J/m^2^) was used for inducing photoadducts and to monitor nucleotide excision repair (NER) activity.

#### Positive and negative assay controls

In the alkaline comet assay, DMSO (1% DMSO for 4 h)-treated TK6 cells were used as negative control and methyl methanesulfonate (100 µM MMS for 4 h)-treated TK6 cells were used as positive control. In the FPG comet assay, water (1% water for 1 h) treated TK6 cells were used as negative control and potassium bromate (2 mM KBrO_3_ for one hour) treated TK6 cells were used as positive control. After treatment, TK6 cells were diluted in freezing medium (RPMI 1640 containing 10% fetal calf serum, 1% _L_-glutamin and 10% DMSO) with a cell density of 1 × 10^6^/ml and aliquoted in 250 µl volumes. These aliqouts were frozen in a freezing container with isopropanol at − 80 °C overnight and stored at − 80 °C. On the day of experiment, one aliquot from each negative and positive control were thawed rapidly and then without washing steps placed on ice. The percentage of DNA in the tail from these methodological negative and positive controls is listed in Table 1.

**Table 1:** The percentage of DNA in tail from methodological negative and positive control

	DNA in tail (%) (Mean ± SD, *n* = 4)	Significance
1% DMSO	4.92 ± 1.37	
100 µM MMS	8.18 ± 1.35	* *p* ≤ 0.05 vs. 1% DMSO
1% Water	5.31 ± 0.72 (Buffer control) 9.43 ± 1.93 (FPG enzyme)	
2 mM KBrO_3_	4.82 ± 0.62 (Buffer control) 23.19 ± 8.41 (FPG enzyme)	* *p* ≤ 0.05 KBrO_3 FPG enzyme_ vs. KBrO_3 Buffer control_ and 1% water _FPG enzyme_

#### Alkaline comet assay

5 µl of undiluted whole blood or buffy coat were mixed with 200 µl of pre-warmed low melting point agarose (0.8%) at 37 °C. For PBMCs, 50 µl of cell suspension were used for mixing with 120 µl of pre-warmed low melting point agarose (0.8%). 5 µl of these mixtures were used for the preparations of each minigel on a Gelbond film as matrix. After solidification of the minigels, Gelbond films were dipped into a cold lysis solution (1% Triton X-100, 10% dimethyl sulfoxide and 89% lysis buffer containing 10 mM Tris, 2.5 M NaCl and 100 mM Na_2_EDTA with pH 10) for an hour. After lysis, Gelbond films were placed in a horizontal electrophoresis chamber filled with cold alkaline buffer and incubated for 20 min in the dark for DNA unwinding and then electrophoresis was performed (1 V/cm, 20 min). For neutralization, Gelbond films were washed in PBS and then in bidistilled water each for 10 min. For dehydration of minigels, Gelbond films were placed in 70% ethanol for 15 min and then in 100% ethanol for 30 min. After air-drying, Gelbond films were cut into small portions (size of a microscope slide) and stained with GelRed for scoring. The percentage of DNA in tail was scored using Komet6 software in 100 random nuclei per sample (50 nuclei/minigel).

#### FPG comet assay

All steps until lysis were done as described above ("Alkaline comet assay" section). After lysis, Gelbond films were washed three times 10 min in FPG enzyme reaction buffer (40 mM Hepes, 0.1 M KCl, 0.5 mM EDTA and 0.2 mg/ml BSA, pH 8) and then either incubated with FPG enzyme (1.6 µg protein / ml) or with FPG enzyme reaction buffer for an hour at 37 °C in a jar. After this, alkaline unwinding and the following steps were done as described above ("Alkaline comet assay" section).

#### Repair comet assay

Isolated PBMCs were resuspended in RPMI 1640 medium supplemented with 10% fetal calf serum, 1% _L_-glutamin and then split in a 48-well plate (400.000 cells/well) for treatment. A sub-group of the cryopreserved samples was used for testing an alternative protocol with mitogen stimulation. For this, PBMCs were split in a 48-well plate and then either incubated with phytohaemaglutinine (PHA, 2.4 µg/ml) or without PHA for 16 h at 37 °C. On the next day, these samples were treated as described and comet assay was performed. To determine BER activity, some of the wells were treated with 40 µM of H_2_O_2_ for 5 min on ice and to determine NER activity, some of the wells were treated with UVC (5 J/m^2^) either in the presence or in the absence of DNA polymerase inhibitor aphidicolin (APC, 5 µM). After treatment, cells were used either immediately or after a repair time (1 h for BER and two hours for NER at 37 °C). Then comet assays were performed as described above ("Alkaline comet assay" section).

#### Vitality test

A dye exclusion/activation fluorescent vitality test was performed to determine the percentage of viable cells directly after thawing cryopreserved PBMCs and either after 16 h PHA stimulation or after 16 h recovery. For this, 70 µl of cell suspension was mixed with 30 µl of staining solution (2 µl of GelRed stock solution and 12 µl of fluorescein diacetate (FDA, 5 mg/ml in acetone) in 2 ml PBS). 15 µl of this mixture were placed on a glass slide and covered with a cover slip (21 × 26 mm). 200 cells were counted at 200-fold magnification with an Eclipse 55i microscope. The proportion of green cells (vital) to red cells (dead) was evaluated.

#### Statistics

Statistical analysis was done using GraphPad Prism 8.4.2 software. Data were presented as Mean ± SD. Two-way ANOVA Tukey`s multiple comparisons test was applied to confirm the significant differences between the groups. Results were considered significant with *p* ≤ 0.05.

## Results

**Fig. 1:**
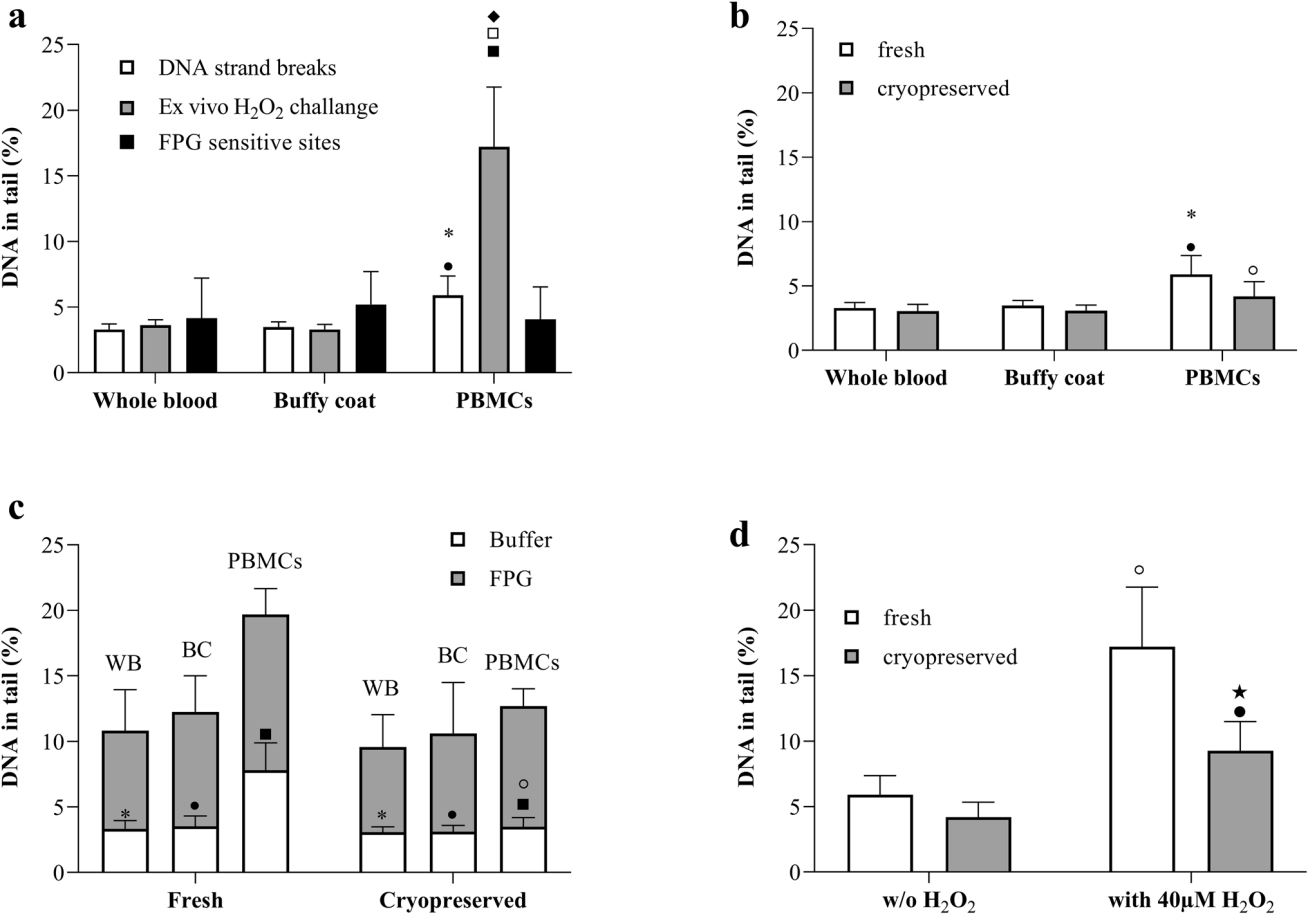
A comparison on basal level of DNA damage in three different blood preparations and the effect of cryopreservation on DNA damage. **a** Basal level of DNA damage among three different blood preparations in alkaline comet (*n* = 20) and FPG comet assay (*n* = 20 for whole blood and buffy coat, *n* = 10 for PBMCs) as well as after an ex vivo hydrogen peroxide challenge (*n* = 10 for whole blood and buffy coat, *n* = 20 for PBMCs). Basal DNA strand breaks: *p ≤ 0.05 vs. whole blood, ● *p* ≤ 0.05 vs. buffy coat and ■ *p* ≤ 0.05 vs. PBMCs. Ex vivo H_2_O_2_ challenge: ♦ *p* ≤ 0.05 vs. whole blood and □*p* ≤ 0.05 vs. buffy coat. **b** The effect of cryopreservation on basal level of DNA strand breaks in three different blood preparations (*n* = 20). Fresh: *p ≤ 0.05 vs. whole blood and ● *p* ≤ 0.05 vs. buffy coat. Cryopreserved: ◦*p* ≤ 0.05 vs. PBMCs. **c** The effect of cryopreservation on basal level of DNA oxidation damage in three different blood preparations (n = 20 for whole blood and buffy coat, *n* = 10 for PBMCs). Buffer (for fresh and cryopreserved): *p ≤ 0.05 vs. whole blood, ● *p* ≤ 0.05 vs. buffy coat and ■ *p* ≤ 0.05 vs. PBMCs. Cryopreserved (only for Buffer): ◦*p* ≤ 0.05 vs. PBMCs. **d** The effect of cryopreservation on hydrogen peroxide-induced DNA strand breaks in PBMCs (*n* = 20). ◦*p* ≤ 0.05 vs. Buffer-fresh, ● *p* ≤ 0.05 vs. Buffer-cyropreserved and ★ *p* ≤ 0.05 vs. FPG-fresh

When fresh samples were used, whole blood and buffy coat preparations exhibited similar amounts of basal DNA strand breaks, whereas PBMCs showed slightly but significantly higher basal DNA strand breaks (Fig. 1a). The percentage of DNA in tail in PBMCs increased significantly upon an ex vivo H_2_O_2_ treatment, but whole blood and buffy coat preparations did not react to this agent. The use of FPG enzyme lead to increased detectable amounts of DNA breaks in all sample types, and the net FPG sensitive sites (damage after FPG treatment minus basal damage) showed a similar level in all three blood preparations.

In Fig. 1b, the comparison of basal DNA strand breaks between fresh and cryopreserved preparations revealed that there was no significant difference between fresh and frozen in whole blood, buffy coat and PBMCs. The FPG comet assay yielded a similar trend for all three blood preparations, but slightly reduced effects in cryopreserved compared to fresh samples (Fig. 1c).

Ex vivo H_2_O_2_ treated fresh and cryopreserved PBMCs yielded a significant increase compared the basal DNA strand breaks. However, the induction in cryopreserved PBMCs was less than in fresh PBMCs (Fig. 1d).

**Fig. 2:**
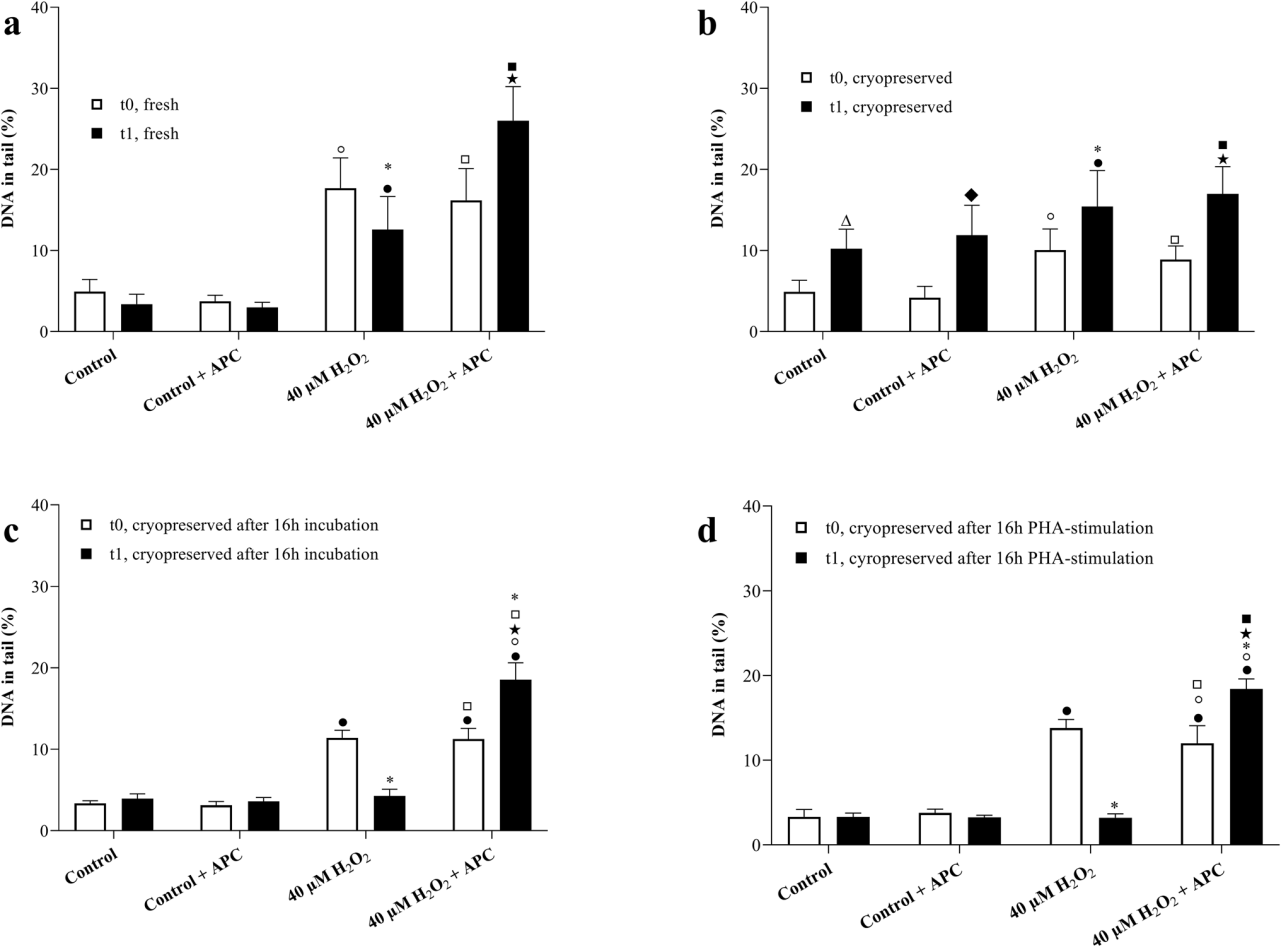
The results of aphidicolin block base excision repair (BER) comet assay. **a** BER activity in freshly isolated PBMCs (*n* = 20). **b** BER activity in cryopreserved PBMCs (*n* = 20). **c** BER activity in cryopreserved PBMCs after 16 h recovery (*n* = 6). **d** BER activity in cryopreserved PBMCs after 16 h PHA stimulation (*n* = 6). t_0_: directly after treating and t_1_: an hour repair. ● *p* ≤ 0.05 vs. Control_t0_, ◦*p* ≤ 0.05 vs. Control _t1_, * *p* ≤ 0.05 vs. 40 µM H_2_O_2 t0_, *p* ≤ 0.05 vs. 40 µM H_2_O_2_ + APC _t0_, □ *p* ≤ 0.05 vs. Control + APC _t0_, ■ *p* ≤ 0.05 vs. Control + APC _t1_, ∆ *p* ≤ 0.05 vs. Control _t0_ and ♦ *p* ≤ 0.05 vs. Control + APC _t0_

Since cryoprotectant had only been added to PBMCs during freezing this was the only sample type that could be investigated for repair activity. In the BER comet assay, 40 µM of H_2_O_2_ was used to induce oxidative DNA damage (Fig. 2). Following the H_2_O_2_ treatment, PBMCs were either used directly (t0) in the comet assays or kept for one hour (t1) at 37 °C to allow repair. During the repair time, 5 µM of aphidicolin was applied to inhibit the DNA polymerase activity preventing filling of repair-mediated gaps. Hydrogen peroxide yielded a significant increase in DNA damage compared to control. The applied aphidicolin concentration did not cause any increase in DNA damage itself. After 1 h repair time and without addition of aphidicolin, a nonsignificant but clear reduction in DNA damage induced by H_2_O_2_ can be seen in Fig. 2a. After one hour repair time, H_2_O_2_ and aphidicolin treated cells showed a significant increase in DNA damage, which represents BER activity. Cryopreserved PBMCs yielded very similar results at t0 (used immediately after treatment; Fig. 2b). However, at t_1_ (after one hour repair time), cryopreserved PBMCs showed significantly elevated DNA damage in all treated cultures independent of the type of treatment. After 16 h of mitogen stimulation or 16 h recovery time, cryopreserved PBMCs yielded similar results to fresh samples in the BER comet assay (Fig. 2c, d). The increase in basal DNA damage had completely disappeared following 16 h incubation (either with or w/o PHA stimulation) and it was possible to measure BER activity following to H_2_O_2_ insult.

**Fig. 3:**
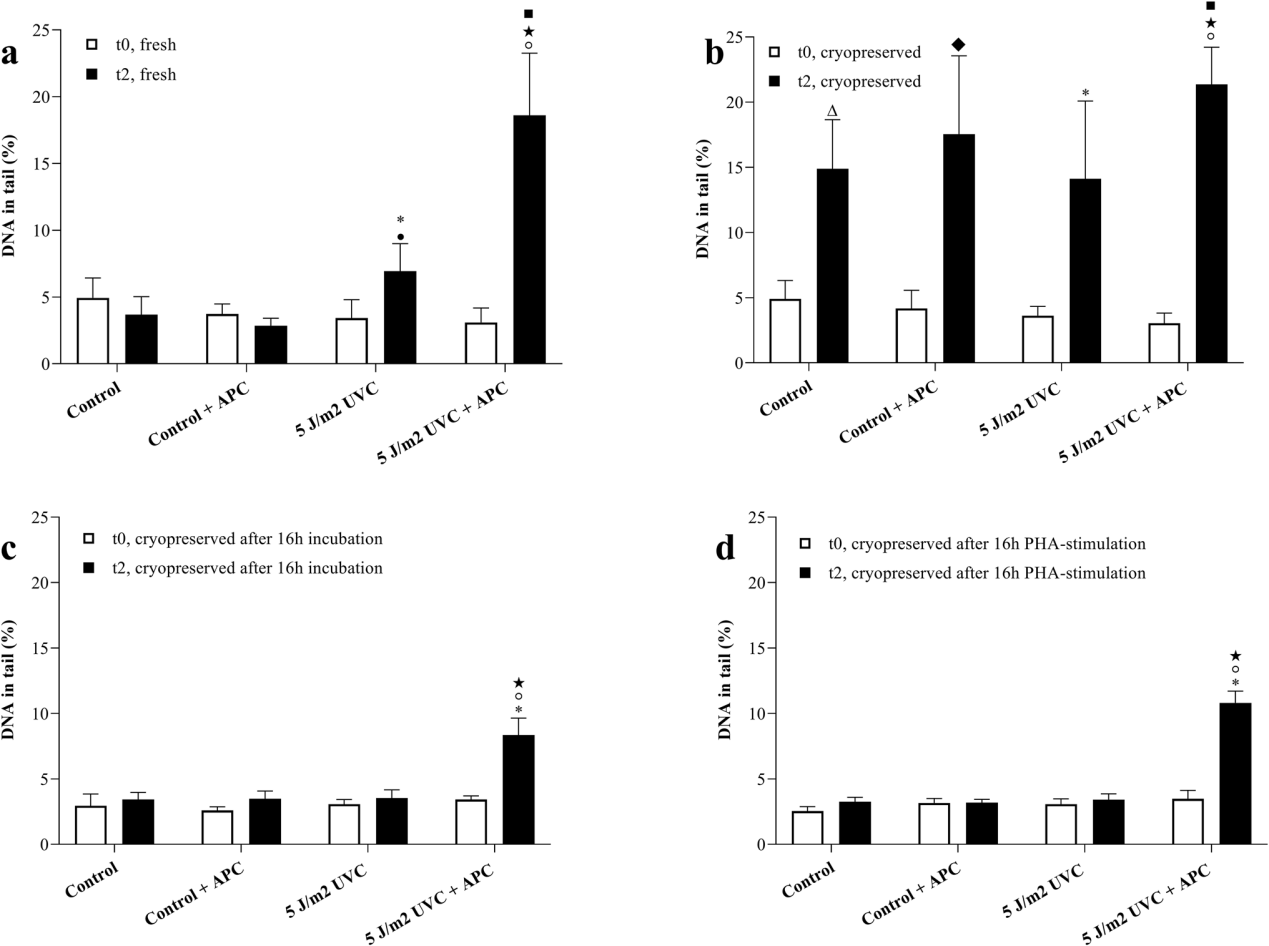
The results of aphidicolin block nucleotide excision (NER) repair comet assay. **a** NER activity in freshly isolated PBMCs (*n* = 20). **b** NER activity in cryopreserved PBMCs (*n* = 20). **c** NER activity in cryopreserved PBMCs after 16 h recovery (*n* = 6). **d** NER activity in crypreserved PBMCs after 16 h PHA stimulation (*n* = 6). t_0_: directly after treating and t_2_: 2 h repair. ◦*p* ≤ 0.05 vs. Control + APC_t2_, * *p* ≤ 0.05 vs. 5 J/m^2^ UVC _t0_, *p* ≤ 0.05 vs. 5 J/m^2^ UVC + APC _t0_, ■ *p* ≤ 0.05 vs. Control + APC _t2_, ∆ *p* ≤ 0.05 vs. Control _t0_ and ♦ *p* ≤ 0.05 vs. Control + APC _t0_

In the NER comet assay, 5 J/m^2^ UVC was used to induce photo adducts in fresh or cryopreserved PBMCs either in presence or in absence of aphidicolin (Fig. 3). Then, the comet assay was performed either directly after treatment at t_0_ or after a two hours repair time at t_2_. The applied dose of UVC did not yield a significant increase in DNA strand breaks at t_0_, but upon repair (removal of photoadducts by NER) at t_2_ lead to a significant increase in DNA damage (Fig. 3a). This increase in DNA damage was higher in the co-treatment group with aphidicolin, which represents the NER incision activity because aphidicolin prevents filling of the NER-induced gaps. Again, similar to what was observed before in the BER variant of the repair comet assay, cryopreserved PBMCs showed a strong increase in basal DNA damage in all treatments after 2 h repair time (Fig. 2b). However, a 16 h recovery time with or without mitogen stimulation prevented the increase in basal damage and made it possible to work with cryopreserved samples for measuring DNA repair activity (Figs. 2c, d, 3c, d).

A dye exclusion/activation vitality test was performed in parallel to the repair comet assays. The percentage of vital cells was determined before and after 16 h incubation (either with or without PHA). In addition, the number of PBMCs was determined using a cell counting chamber following the 16 h incubation. There was no significant difference in the percentage of vital cells after 16 h incubation neither with nor without PHA stimulation. However, the numbers of PBMCs were altered after 16 h. After 16 h of mitogen stimulation, the PBMC number was reduced by 40.80% compared to the number of seeded cells. When 16 h recovery time was applied, the cell number was reduced by 25.91% (Table 2).

**Table 2:** The percentage of vital cells and reduction in the number of PBMCs after thawing directly, after 16 h recovery and after 16 h PHA stimulation

	Vital cells (%) (Mean ± SD, *n* = 6)	Reduction in the number of PBMCs (%)
Directly after thawing	93.21 ± 2.63	
Cryopreserved after 16 h recovery	87.00 ± 5.87	25.91 ± 14.60
Cryopreserved after 16 h PHA stimulation	89.45 ± 3.85	40.80 ± 16.40

## Discussion

Due to its several modified versions, the comet assay is not only used to determine DNA strand breaks, but also to investigate the type of DNA lesions, antioxidant status of samples and their DNA repair activity in human biomonitoring studies. Performing an enhanced throughput version of the comet assay with large sample numbers can increase the quality of this assay for human biomonitoring, because many samples can be run in the same electrophoresis, eliminating possible minor day to day method variability. However, this will usually require collecting samples over time, as it is also done in biobanks. Whether the use of such cryopreserved samples is suitable for various modifications of the comet assay was tested here. We compared different blood preparations (whole blood, buffy coat and isolated PBMCs) and the effect of cryopreservation on DNA damage and DNA repair activity. Our findings showed a similar basal level of DNA damage in all fresh and cryopreserved blood preparations, with PBMCs exhibiting slightly higher levels than whole blood and buffy coat. The basal level of oxidized DNA lesions, represented as Net FPG sensitive sites, was also similar for all blood preparations, and either fresh or cryopreserved. Ladeira et al. (2019), have previously compared the basal DNA strand breaks from 3 blood samples with the same 3 different blood preparation types as here, also fresh and cryopreserved and our results using 20 samples are in complete agreement, further supporting their findings.

Furthermore, we used an exogenous insult to determine the sensitivity of different blood preparations against an oxidizing agent. Hydrogen peroxide treatment yielded a significant increase in DNA damage in PBMCs, but not in whole blood and buffy coat preparations. This is in agreement with several publications, which compared the sensitivity of whole blood and isolated mononuclear cells and showed that the whole blood preparations were resistant to exogenous H_2_O_2_ treatment (Akor-Dewu et al. 2014; Chuang and Hu 2004; Giovannelli et al. 2003). There was no significant difference between fresh and cryopreserved PBMCs regarding their response to the exogenous H_2_O_2_ insult. Ladeira et al. (2019), investigated the influence of exogenous hydrogen peroxide treatment as well and different from our results, buffy coat also exhibited a slight increase. They discussed a possible protective effect of erythrocyte and plasma fractions against oxidizing agents due to cellular antioxidant components such as catalase enzyme and glutathione (Ladeira et al. 2019). If that is the case, differences in the preparation of buffy coat might explain the slightly different results. Buffy coat preparations have a different composition compared to whole blood and PBMCs. They do contain erythrocytes, but to a lower degree than whole blood. On the other hand, buffy coat samples contain a higher percentage of neutrophils than the PBMC fractions. These differences might explain their lower sensitivity compared to the PBMC preparation and slightly higher sensitivity compared to whole blood regarding oxidizing agents. Andreoli et al. (1999), treated whole blood and isolated white blood cells with methyl methanesulfonate (MMS) and H_2_O_2_. They observed a significant increase in DNA damage in both sample types after MMS treatment, but not with H_2_O_2_, which only led to an elevated DNA damage in white blood cells, but not in whole blood cells, similar to our and Ladeira et al. (2019) observations.

Our findings support the study from Al-Salmani et al. (2011), by showing once more that cryoprotection is not necessary for the storage of whole blood as well as buffy coat preparations. This is a great advantage if human biomonitoring studies are performed with large sample numbers and it can reduce the time required for sample preparation significantly.

The FPG comet assay was performed with fresh and cryopreserved blood preparations. All three different blood preparations yielded a similar outcome in the FPG comet assay with fresh and cryopreserved samples. Net FPG sensitive sites were not different between the different blood preparations or between fresh and cryopreserved samples. Thus, all three preparations were found suitable for performing the FPG comet assay.

The comet assay is mostly used to determine DNA damage, but there are different modified versions of comet assay, which allow detection of the DNA repair activity. We performed BER and NER comet assays after inducing specific lesions using either H_2_O_2_ or UVC. Freshly isolated PBMCs or cryopreserved PBMCs were used in both variants. Both repair assays yielded clear results in freshly isolated PBMCs. However, in cryopreserved PBMCs, the repair duration lead to a significant increase in basal DNA damage and no result pattern revealing DNA repair activity was detectable. The NER comet assay has been used in freshly isolated white blood cells by different working groups. For example, freshly isolated white blood cells were treated with benzo[a]pyrene diol epoxide and both studies (Speit et al. 2013; Vande Loock et al. 2010) were able to determine NER incision activity. There are a limited number of studies, which performed repair comet assays with cryopreserved PBMCs (Table 3). The summarized studies in Table 3 reveal huge variations in the protocol for the DNA repair comet assay with cryopreserved blood preparations. Recovery time, PHA stimulation or use of APC are inconsistent among the labs and their effect on the outcome is not well understood.

**Table 3:** Studies that performed repair comet assay with cryopreserved blood preparations

Type of sample	Recovery after thawing	PHA stimulation	Conditions of repair assay	Observations	References
Fresh and cryopreserved PBMCs (stored at − 70 °C)	No	Only for cryopreserved PBMCs for 2 h during repair	The alkaline comet assay was used either directly after treating with H_2_O_2_ or after Ɣ-irradiation or after 2 h repair	No difference between fresh and cryopreserved PBMCs in their response to insults or DNA repair activity	Visvardis et al. (1997)
Fresh and cryopreserved PBMCs (stored for 24 h and 2 months at − 80 °C)	No	No	The alkaline comet assay was performed either directly after treatment with H_2_O_2_ or after repair (4, 8 and 24 h)	Incubation of cryopreserved PBMCs at 37 °C after thawing caused increased DNA damage and the cryopreserved PBMCS were not able to repair	Duthie et al. (2002)
Fresh and cryopreserved PBMCs (for 1 and 3.5 h at − 80 °C)	Yes, overnight recovery for both cell preparations and then 30 min recovery after agarose embedding	No	Agarose embedded cells were irradiated (Ɣ-irradiation), and alkaline comet assay was performed at 0, 5, 15, 30 and 60 min	Cryopreserved cells showed slower DNA repair activity	Trzeciak et al. (2008)
Fresh and cryopreserved PBMCs	Yes, 24 h for both sample types	Yes, 24 h for both sample types	The APC-block NER comet assay was performed after treating with benzo[a]pyrene diol epoxide for 2 h	There was a significant correlation between the DNA repair capacity of fresh and cryopreserved PBMCs	Allione et al. (2012)
Fresh and cryopreserved PBMCs (stored for one week up to one year in liquid nitrogen)	One group of cryopreserved cells was used directly after thawing and another one after 4 h recovery	No	The alkaline comet assay was performed to analyse the repair activity directly after H_2_O_2_ treatment and after 1 h repair with or w/o APC	No difference was observed due to the freeze/thaw cycle	Odongo et al. (2019)
Fresh whole blood, cryopreserved whole blood and cryopreserved PBMCs (at least a month at − 80 °C)	Yes, 24 h for all three sample types	Yes, 24 h for all three sample types	The alkaline comet assay was performed either directly after treating with bleomycin/UVC/MMS or at three different repair times	There was no difference between fresh and cryopreserved samples except the repair of MMS induced damage, which was better in fresh samples	Valdiglesias et al. (2020)

Consistent with our findings, Duthie et al. (2002), showed that incubating cryopreserved PBMCs at 37 °C for 4 h or more caused a significant increase in the basal DNA damage level. The PHA-stimulated PBMCs had a higher DNA repair capacity compared to the unstimulated ones. Half of the published works used a recovery time of 16 or more hours, while the other half worked with freshly thawed or shortly recovered PBMCs. In addition, half of the studies employed mitogen stimulation. PHA activates lymphocytes to re-enter the cell cycle (and later to go through mitosis). One of the studies, (Barret et al. 1995), investigated the NER incision activity with various methods in PHA-stimulated and unstimulated peripheral blood lymphocytes and found a significant increase of incision activity upon PHA-treatment. The choice of a long recovery period might result from following the successful protocol of others or own negative observations with freshly thawed PBMC. However, this has not been reported clearly so far. Since the analysis of DNA repair activity requires cell culture, viability of cells after thawing has crucial importance. The dying cells after thawing can lead to elevated DNA in tail and cause an increased background as we observed. If one compares DNA damage at 16 h with that at a few hours (e.g. 1–4) after thawing, the reduction of damage might be misinterpreted as DNA repair while it really represents the death of highly damaged cells. Since repair protocols might call for certain treatment durations, it is important to keep this in mind and record cell numbers in addition to DNA damage when working with frozen samples.

Since a method variant with preparing extracts containing repair enzymes from cryopreserved samples and using the extracts on other cells in the comet assay works well (Gaivão et al. 2009; Langie et al. 2010; Paz-Elizur et al. 2007), it is unlikely that cryopreservation and/or thawing have an influence on the activity of repair enzymes. Instead, this observed increase in % tail DNA within the first hours after thawing might be related to the membrane integrity of the thawed PBMCs, for example by causing a release of nucleases from intracellular organelles. In our experiments, PHA stimulation of cryopreserved PBMCs yielded a detectable repair activity after 16 h, but only 60% of cryopreserved cells were still present at the time of fixation after stimulation. After 16 h recovery time without PHA stimulation, we were also able to analyse repair activity in cryopreserved PBMCs, but only 75% of cryopreserved cells were still present.

In summary, cryopreserved whole blood, buffy coat and PBMCs can be used to analyse the DNA damage in the comet assay. Whole blood and buffy coat preparations do not require any addition of cryoprotectant, which makes the freezing procedure simple and increases the viability of cells after thawing. However, cryopreserved PBMCs offer the additional possibility of an ex vivo induction of DNA lesions. For example, an ex vivo hydrogen peroxide challenge can be a useful approach to compare the antioxidant status of subjects in nutritional studies. In contrast to the successful utilization of cryopreserved samples in the alkaline and FPG comet assay, it was only possible to use cryopreserved PBMCs for NER or BER comet assay with an introduction of a recovery time before use. Thus, to analyse DNA repair capacity in cryopreserved PBMCs, a 16 h recovery time (with or without PHA) is recommended. However, during the recovery, a considerable reduction of cell number may be observed, and it should be considered for each study whether that is acceptable for the aim of the study, since selective loss of certain cells cannot be excluded currently. In studies reporting repair kinetics of freshly thawed cells over a longer time, a concurrent cell count is needed.

## References

[CR1] Akor-Dewu MB, El Yamani N, Bilyk O, Holtung L, Tjelle TE, Blomhoff R, Collins AR (2014). Leucocytes isolated from simply frozen whole blood can be used in human biomonitoring for DNA damage measurement with the comet assay. Cell Biochem Funct.

[CR2] Al-Salmani K (2011). Simplified method for the collection, storage, and comet assay analysis of DNA damage in whole blood. Free Radic Biol Med.

[CR3] Allione A (2012). Validation of the nucleotide excision repair comet assay on cryopreserved PBMCs to measure inter-individual variation in DNA repair capacity. Mutagenesis.

[CR4] Andreoli C, Rossi S, Leopardi P, Crebelli R (1999). DNA damage by hydroquinone in human white blood cells: analysis by alkaline single-cell gel electrophoresis Mutation Research/Genetic Toxicology and Environmental. Mutagenesis.

[CR5] Azqueta A, Gutzkow KB, Priestley CC, Meier S, Walker JS, Brunborg G, Collins AR (2013). A comparative performance test of standard, medium- and high-throughput comet assays. Toxicol In Vitro.

[CR6] Azqueta A (2020). Application of the comet assay in human biomonitoring: an hCOMET perspective. Mutat Res.

[CR7] Azqueta A (2019). DNA repair as a human biomonitoring tool: comet assay approaches. Mutat Res/Rev Mutat Res.

[CR8] Bankoglu EE, Gerber J, Kodandaraman G, Seyfried F, Stopper H (2020). Influence of bariatric surgery induced weight loss on oxidative DNA damage Mutation Research/Genetic Toxicology and Environmental. Mutagenesis.

[CR9] Bankoglu EE (2018). Reduction of DNA damage in peripheral lymphocytes of obese patients after bariatric surgery-mediated weight loss. Mutagenesis.

[CR10] Barret J-M, Calsou P, Salles B (1995). Deficient nucleotide excision repair activity in protein extracts from normal human lymphocytes. Carcinogenesis.

[CR11] Bonassi S (2007). An increased micronucleus frequency in peripheral blood lymphocytes predicts the risk of cancer in humans. Carcinogenesis.

[CR12] Chuang C-H, Hu M-L (2004). Use of whole blood directly for single-cell gel electrophoresis (comet) assay in vivo and white blood cells for in vitro assay. Mutat Res/Genetic Toxicol Environ Mutagen.

[CR13] Collins AR, Azqueta A (2012a) Chapter 4—single-cell gel electrophoresis combined with lesion-specific enzymes to measure oxidative damage to DNA. In: Conn PM (ed) Methods in cell biology, vol 112. Academic Press, Cambridge, pp 69–92. 10.1016/B978-0-12-405914-6.00004-4

[CR14] Collins AR, Azqueta A (2012). DNA repair as a biomarker in human biomonitoring studies; further applications of the comet assay. Mutat Res/Fund Mol Mech Mutagen.

[CR15] Duthie SJ, Pirie L, Jenkinson AM, Narayanan S (2002). Cryopreserved versus freshly isolated lymphocytes in human biomonitoring: endogenous and induced DNA damage, antioxidant status and repair capability. Mutagenesis.

[CR16] Gaivão I, Piasek A, Brevik A, Shaposhnikov S, Collins AR (2009). Comet assay-based methods for measuring DNA repair in vitro; estimates of inter- and intra-individual variation. Cell Biol Toxicol.

[CR17] Giovannelli L, Pitozzi V, Riolo S, Dolara P (2003). Measurement of DNA breaks and oxidative damage in polymorphonuclear and mononuclear white blood cells: a novel approach using the comet assay. Mutat Res/Genet Toxicol Environ Mutagen.

[CR18] Gutzkow KB, Langleite TM, Meier S, Graupner A, Collins AR, Brunborg G (2013). High-throughput comet assay using 96 minigels. Mutagenesis.

[CR19] Koppen G, De Prins S, Jacobs A, Nelen V, Schoeters G, Langie SAS (2017). The comet assay in human biomonitoring: cryopreservation of whole blood and comparison with isolated mononuclear cells. Mutagenesis.

[CR20] Ladeira C, Koppen G, Scavone F, Giovannelli L (2019). The comet assay for human biomonitoring: effect of cryopreservation on DNA damage in different blood cell preparations. Mutat Res/Genet Toxicol Environ Mutagen.

[CR21] Langie SA, Wilms LC, Hämäläinen S, Kleinjans JC, Godschalk RW, van Schooten FJ (2010). Modulation of nucleotide excision repair in human lymphocytes by genetic and dietary factors. Br J Nutr.

[CR22] Langie SAS (2015). Causes of genome instability: the effect of low dose chemical exposures in modern society. Carcinogenesis.

[CR23] Milić M, Ožvald I, Vinković Vrček I, Vučić Lovrenčić M, Oreščanin V, Bonassi S, Del Castillo ER (2019). Alkaline comet assay results on fresh and one-year frozen whole blood in small volume without cryo-protection in a group of people with different health status. Mutat Res/Genet Toxicol Environ Mutagen.

[CR24] Møller P, Stopper H, Collins AR (2020). Measurement of DNA damage with the comet assay in high-prevalence diseases: current status and future directions. Mutagenesis.

[CR25] Odongo GA, Skatchkov I, Herz C, Lamy E (2019). Optimization of the alkaline comet assay for easy repair capacity quantification of oxidative DNA damage in PBMC from human volunteers using aphidicolin block. DNA Repair (Amst).

[CR26] Paz-Elizur T (2007). Development of an enzymatic DNA repair assay for molecular epidemiology studies: distribution of OGG activity in healthy individuals. DNA Repair.

[CR27] Speit G, Leibiger C, Kuehner S, Högel J (2013). Further investigations on the modified comet assay for measuring aphidicolin-block nucleotide excision repair. Mutagenesis.

[CR28] Trzeciak AR, Barnes J, Evans MK (2008). A modified alkaline comet assay for measuring DNA repair capacity in human populations. Radiat Res.

[CR29] Valdiglesias V, Sánchez-Flores M, Fernández-Bertólez N, Au W, Pásaro E, Laffon B (2020). Expanded usage of the Challenge-Comet assay as a DNA repair biomarker in human populations: protocols for fresh and cryopreserved blood samples, and for different challenge agents. Arch Toxicol.

[CR30] Vande Loock K, Decordier I, Ciardelli R, Haumont D, Kirsch-Volders M (2010). An aphidicolin-block nucleotide excision repair assay measuring DNA incision and repair capacity. Mutagenesis.

[CR31] Visvardis EE, Tassiou AM, Piperakis SM (1997). Study of DNA damage induction and repair capacity of fresh and cryopreserved lymphocytes exposed to H2O2 and γ-irradiation with the alkaline comet assay. Mutat Res/DNA Repair.

